# Differential diagnosis between pemphigoid and erosive lichen planus

**DOI:** 10.1590/1678-7757-2021-0657.let

**Published:** 2022-04-20

**Authors:** Zhe Cheng, Jianqiu Jin

**Affiliations:** 1 Beijing Hospital National Center of Gerontology Department of Stomatology Beijing China Beijing Hospital, National Center of Gerontology, Department of Stomatology; Chinese Academy of Medical Sciences, Institute of Geriatric Medicine, Beijing, China.

Dear Editor

We read an interesting article published by Saglam, et al.^1^ (2021) titled “Efficacy of injectable platelet-rich fibrin in the erosive oral lichen planus a split-mouth, randomized, controlled clinical trial.” The authors used platelet-rich fibrin injections to treat erosive oral lichen planus and compared it to other gums injected with steroid hormones on the other side of the oral cavity. Platelet-rich fibrin injection was found to achieve good results.^1^ The pictures of the case were diagnosed as erosive oral lichen planus, which may be inaccurate.

**Figure 1:**
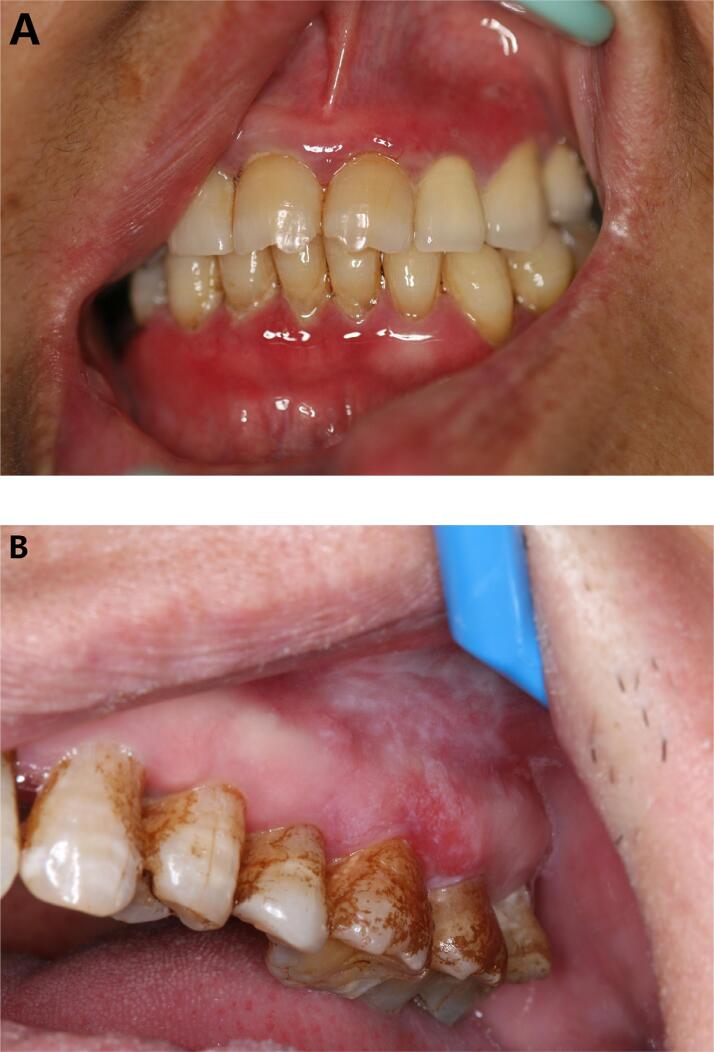
(A) Moderate desquamative gingivitis of pemphigoid; (B) Lichen planus gingivalis of posterior teeth showed white reticular striations

Pemphigoid is a rare autoimmune bullous disease that affects mucous membranes and often occurs in the oral mucosa and conjunctiva. Diseases can occur in any part of the oral mucosa, and the gums are the first and most often damaged parts. Typical manifestations are erythema, blistering, erosion, and surfaces covered with a false membrane. In the early stages of damage, local congestion and redness occur at the gingival margin and the attached gingiva, forming small blisters with a thick blister wall. A gray-white blister membrane was observed after the blister wall was broken, and a smooth red superficial ulcer was observed after the blister wall was removed (Figure 1A). Histopathological examination showed the formation of a subepithelial blister, and immunofluorescence examination showed an emerald-green fluorescent band in the basement membrane. The diagnosis of pemphigoid can be confirmed by clinical manifestation, histopathology, and direct immunofluorescence examination. Circulating autoantibodies against the pemphigoid antigen BP 180 can be detected in serum samples using ELISA and are useful in both diagnosis and monitoring of disease activity.^2^ Therefore, in the presence of exfoliated gums or erythema, pemphigoid should be considered first.

Oral lichen planus can occur in any part of the oral mucosa. Buccal mucosa is the most common site for its occurrence, and its lesions are mostly symmetrically distributed as pinpoint-sized grayish-white papules, which form fine keratinized striations. These gray-white keratinized striations may be accompanied by hyperemia, erosion, atrophy, blisters, and other damages. Lichen planus gingivalis is relatively rare. Gray-white markings can be seen in the attached gingiva, and congestive erythema, edema or even erosion occurs due to epithelial atrophy. Erosive lichen planus is mainly characterized by exfoliative damage to the gums, bright red color, and bleeding when touched.^3^ However, gray-white pearly striations can be seen in the tissues adjacent to the mucosa or other parts of the oral cavity (Figure 1B). Pemphigoid has no white striations. Biopsies of gingival oral lichen planus can mimic mucous membrane pemphigoid, although hydropic basal cell degeneration and colloid bodies can often be seen. Direct immunofluorescence is sometimes needed to distinguish these two lesions.^4^
